# Prolactin Receptor in Primary Hyperparathyroidism – Expression, Functionality and Clinical Correlations

**DOI:** 10.1371/journal.pone.0036448

**Published:** 2012-05-11

**Authors:** Felix Haglund, Ming Lu, Vladana Vukojević, Inga-Lena Nilsson, Adam Andreasson, Mensur Džabić, Robert Bränström, Anders Höög, C. Christofer Juhlin, Catharina Larsson

**Affiliations:** 1 Department of Molecular Medicine and Surgery, Karolinska Institutet, Stockholm, Sweden; 2 Center for Molecular Medicine, Karolinska Institutet, Stockholm, Sweden; 3 Department of Clinical Neuroscience, Karolinska Institutet, Stockholm, Sweden; 4 Department of Medicine-Solna, Karolinska Institutet, Stockholm, Sweden; 5 Department of Oncology-Pathology, Karolinska Institutet, Stockholm, Sweden; Baylor College of Medicine, United States of America

## Abstract

**Background:**

Primary hyperparathyroidism (PHPT) is an endocrine disorder most commonly affecting women, suggesting a role for female hormones and/or their receptors in parathyroid adenomas. We here investigated the prolactin receptor (PRLr) which is associated with tumours of the breast and other organs.

**Methodology/Principal Findings:**

PRLr expression was investigated in a panel of 37 patients with sporadic parathyroid tumours and its functionality in cultured parathyroid tumour cells. In comparison with other tissues and breast cancer cells, high levels of prolactin receptor gene (*PRLR*) transcripts were demonstrated in parathyroid tissues. PRLr products of 60/70 kDa were highly expressed in all parathyroid tumours. In addition varying levels of the 80 kDa PRLr isoform, with known proliferative activity, were demonstrated. In parathyroid tumours, PRLr immunoreactivity was observed in the cytoplasm (in all cases, n = 36), cytoplasmic granulae (n = 16), the plasma membrane (n = 12) or enlarged lysosomes (n = 4). In normal parathyroid rim (n = 28), PRLr was uniformly expressed in the cytoplasm and granulae. In *in vitro* studies of short-term cultured human parathyroid tumour cells, prolactin stimulation was associated with significant transcriptional changes in JAK/STAT, RIG-I like receptor and type II interferon signalling pathways as documented by gene expression profiling. Moreover, *PRLR* gene expression in parathyroid tumours was inversely correlated with the patients’ plasma calcium levels.

**Conclusions:**

We demonstrate that the prolactin receptor is highly abundant in human parathyroid tissues and that PRLr isoforms expression and PRLr subcellular localisation are altered in parathyroid tumours. Responsiveness of PRLr to physiological levels of prolactin was observed in the form of increased PTH secretion and altered gene transcription with significant increase of RIG-I like receptor, JAK-STAT and Type II interferon signalling pathways. These data suggest a role of the prolactin receptor in parathyroid adenomas.

## Introduction

Primary hyperparathyroidism (PHPT) is a common endocrine disorder characterized by increased levels of serum calcium and parathyroid hormone (PTH) and symptoms of e.g. osteoporosis, kidney stones, psychological disturbances or cardiovascular disease [1], [2], [3]. Available reports suggest that the disease affect approximately 1% of the population. In addition, the incidence of PHPT increases in women after menopause [4], where prevalence of 3% have been reported [5]. The molecular background has been elucidated for a large part of familial forms of the disease, caused by constitutional mutations in the multiple endocrine neoplasia type 1 gene (*MEN1*),the hyperparathyroidism-jaw tumour syndrome gene (*HRPT2*) or the calcium sensing receptor gene (*CASR*). However, the molecular background remains unknown for the majority of the common sporadic form of PHPT, caused by a single benign parathyroid tumour. As this group most frequently affects women it is suggested that female hormones and/or their receptors may have a role in these tumours.

Human prolactin, a hormone secreted from the anterior pituitary gland, is known to have multiple functions. In addition to being responsible for lactation and mammary growth, prolactin has calciotropic effects in the intestine, kidney and skeleton [6], [7], [8], [9] and has also been associated with cancer development *e.g.* in the breast and ovary [10], [11], [12], [13], [14], [15], [16]. In pancreatic beta cells, prolactin signalling is known to inhibit the *MEN1* gene product menin, causing β-cell proliferation during pregnancy [17], [18]. The prolactin receptor (PRLr), a type I cytokine receptor, is encoded by the *PRLR* gene in chromosomal region 5p13.2. In humans the five known functional isoforms are encoded by five overlapping transcripts, produced by alternative splicing (Figure S1) [19]. While the long and ΔS1 PRLr isoforms, originally isolated from T47D breast cancer cells, may activate proliferative signalling through the Janus kinase/signal transducers and activators of transcription (JAK/STAT) pathway, the short isoforms IF, S1a and S1b lack the tyrosine rich residue required for full STAT binding [20], [21], [22], [23]. N-glycosylation of the extracellular domain of PRLr is important for membrane localization [24]. The protein kinase GSK3β is a key regulator of PRLr degradation through phosphorylation of the PRLr serine residue at amino acid position 349. In breast cancer cells, inhibition of GSK3β through serine phosphorylation on residue 9 has been shown to correlate with elevated PRLr levels [25].

Some previous observations suggest a role for PRLr in parathyroid cells. High *PRLR* expression in parathyroid adenomas may be identified from previous reports of expression profiling of parathyroid tissues reported by us and others [26], [27], and from expressed sequence tag (EST) profiling (Hs.368587) of parathyroid adenoma (973 TPM) as compared to breast tumour (424 TPM) (http://www.ncbi.nlm.nih.gov/UniGene/ESTProfileViewer.cgi?uglist=Hs.368587). In addition, *PRLR* knockout mice exhibited phenotypic alterations of calcium, PTH levels and bone formation [8], [28]. Finally, prolactin stimulation of bovine parathyroid cells *in vitro* was reported to increase PTH secretion independent of the parathyroid beta-adrenergic and dopaminergic systems [29]. However, clinical observations of the relationship between PTH and prolactin levels are conflicting, and processes *in vivo* such as e.g. tumour induced hyperprolactinemia, pregnancy, the menstrual cycle and polycystic ovary syndrome have proved too complex to establish a consensus model of causality [6], [7], [29], [30], [31], [32], [33], [34], [35], [36], [37], [38], [39].

Given the frequent occurrence of PHPT in women and the role of PRLr in other tumours we aimed to assess PRLr expression and functionality in human parathyroid tumours. We determined *PRLR* gene and PRLr isoform expression in parathyroid tumours and normal tissues, and evaluated parathyroid tumour cell function and expression profiles upon prolactin stimulation *in vitro*.

## Results

### *PRLR* Gene Expression in Parathyroid Tissues

**Figure 1:**
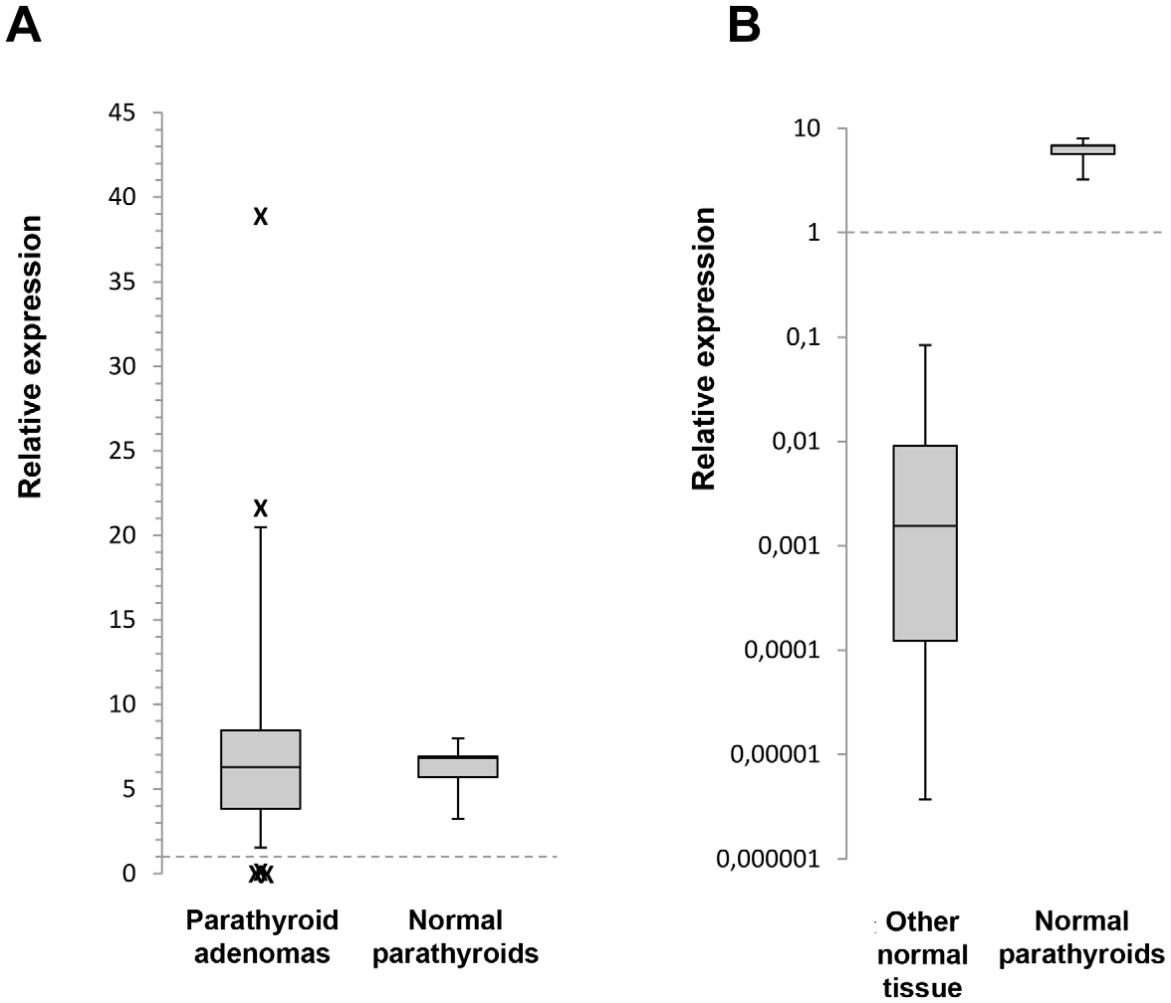
qRT-PCR analysis for *PRLR* expression in normal parathyroid tissue and parathyroid tumours. Box-plots show*PRLR*-total mRNA expression after normalization in normal parathyroid tissue and parathyroid tumours (A), and normal parathyroid and other normal tissues (B). The arbitrary value of 1.0 indicating the expression level in MCF-7 cells is indicated.

Expression of *PRLR* gene transcripts was determined by qRT-PCR analyses of parathyroid tumours and normal tissues. Using the *PRLR*-total assay (detecting LF, ΔS1, IF and S1a but not S1b), the highest levels of expression was observed in parathyroid tissues (Figure 1; Table S3). Non-parathyroid normal tissues had, as expected, variable but lower expression than the MCF-7 cell line, and the highest level of *PRLR*-total was observed in the placenta (Figure S2). Normal parathyroid tissues expressed nearly tenfold higher levels compared to the MCF-7 cell line. *PRLR*-total expression was observed in 35/37 parathyroid tumours, at levels that are higher (>1.5, n = 7), lower (<0.5; n = 9) or comparable (>0.5 and <1.5; n = 19) to the mean level for the normal parathyroids (0.77–1.30). Two additional assays were applied for the detection of transcripts corresponding to LF and ΔS1, showing that their expression levels generally followed the levels of *PRLR-*total in parathyroid tissues (Figure S2A and S3). The *PRLR*-S1a assay, detecting only the S1a transcript, showed that its level was relatively low in MCF-7 (Figure S2B) and within the range of the normal tissues (Figure S3).

Expression of the *PRLR*-ΔS1 transcript, or of all other *PRLR* transcripts (except ΔS1), was demonstrated by regular qualitative RT-PCR, using transcript specific primers and detection of product of expected size by agarose gel electrophoresis. For comparison expression of the other four transcripts *PRLR*-LF/IF/S1a/S1b was similarly demonstrated by RT-PCR.

### Expression of PRLr Isoforms and GSK3β in Parathyroid Tumours

**Figure 2:**
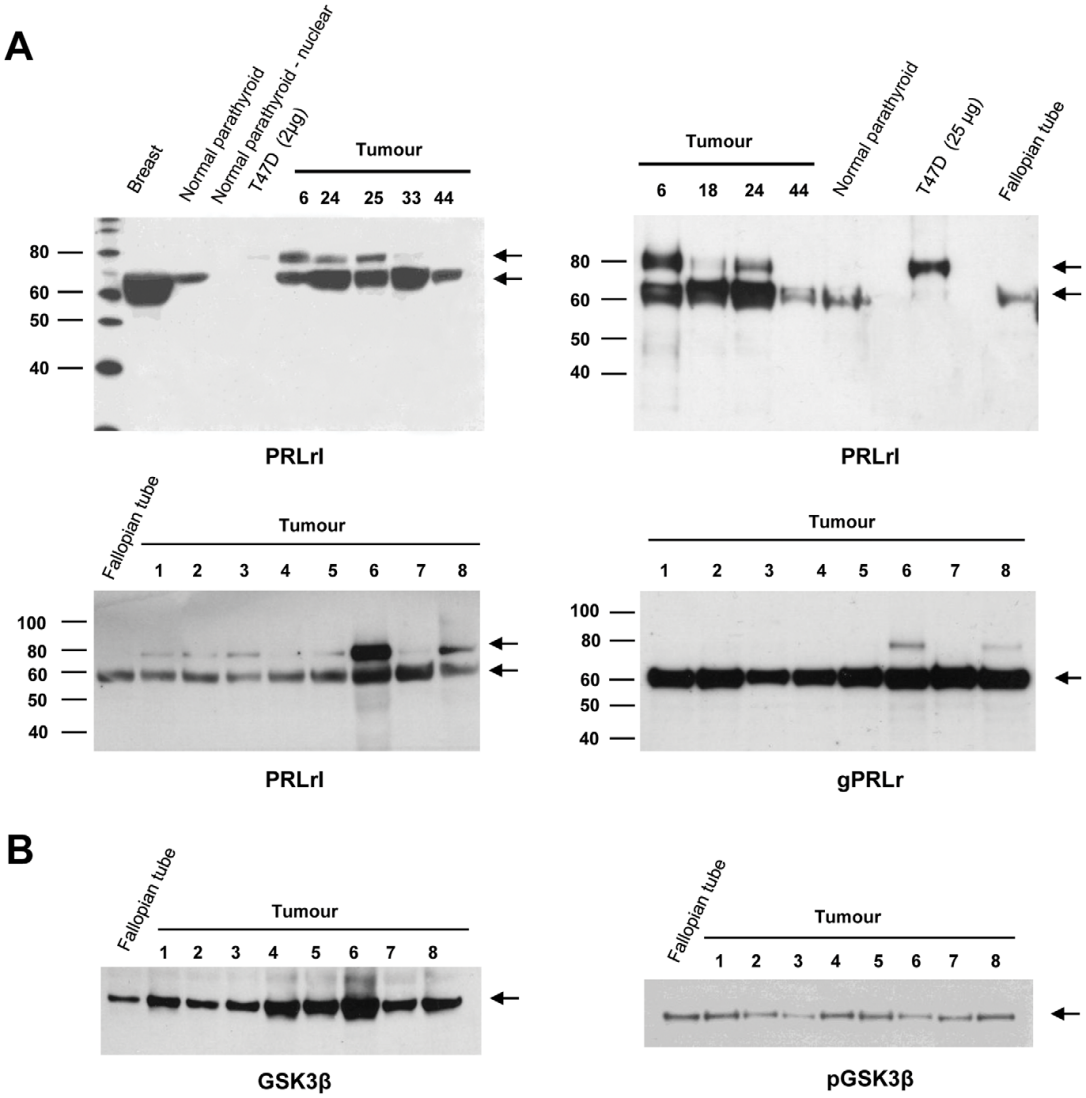
Western blot analysis of protein expression for GSK3β and isoforms of the prolactin receptor. A) Detection of 80 kDa and 60/70 kDa products with the PRLrI antibody in normal tissues, T47D cells and parathyroid tumours, and 60/70 kDa N-glycosylated PRLr products with the gPRLr antibody in parathyroid tumours. B) Expression of total GSK3β (left) as well as Ser9-phosphorylated GSK3β (right) in parathyroid tumours and normal tissue.

The PRLr was expressed in T47D breast cancer cells as an 80 kDa product, corresponding to the long isoform reported by Galskaard et al. [40], while in normal parathyroid, breast and fallopian tube a shorter PRLr product of 60–70 kDa was detected (Figure 2). Nuclear extract of normal parathyroid tissue was negative. PRLr expression was demonstrated by Western blot analysis for both PRLr and the N-glycosylated form. The 60/70 kDa PRLr product was expressed in all 37 parathyroid tumours (Figure 2A; Table S3). In addition the 80 kDa isoform was observed at levels similar to the 60/70 kD PRLr in 10 tumours or weaker in 15 tumours. In 12 tumours the 80 kDa product was not revealed or barely detectable (Table S3). In the normal parathyroid only the 60/70 kDa product was revealed. N-glycosylated PRLr was mainly observed as a product of 60/70 kDa in size, which was detected in all tumours analysed (Figure 2A).

GSK3β is known to phosphorylate ser349 of the long and ΔS1PRLr isoforms, and GSK3β ser9-phosphorylation is needed for PRLr degradation [25]. We therefore analysed GSK3β expression concerning levels of total GSK3β as well as the serine-9 phosphorylated form (Figure 2B; Table S3). 35/37 parathyroid tumours and fallopian tube expressed total GSK3β at comparable levels, while in 2 tumours only weak expression was observed. Ser9-Phosphorylated GSK3β was strongly expressed in 29 parathyroid tumours (Figure 2B), weakly expressed in 6 tumours, and barely detectable in 2 (Table S3). Ser9-phosphorylated GSK3β was not detected in the normal parathyroid gland. As compared to the results for the 80 kDa PRLr product the 2 tumours with barely detectable Ser9-phosphorylated GSK3β lacked the PRLr 80 kDa product (Table S3). Out of the 6 tumours with weak Ser9-phosphorylated GSK3β,3 lacked and one had weak PRLr 80 kDa expression.

### Expression and Subcellular Localization of PRLr in Parathyroid Tumours

**Figure 3:**
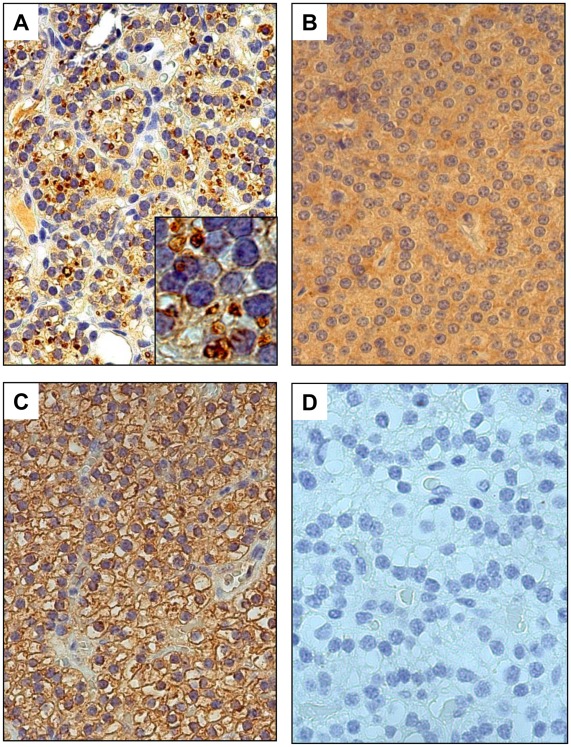
Immunohistochemical analysis of PRLr expression using the PRLrI antibody . The photomicrographs show parathyroid tumour tissues (A–C) and negative control (D). Parathyroid tumours are shown with immunostaining of cytoplasmic granulae and cytoplasm (A), of cytoplasm only (B), and of cell membrane and cytoplasm (C).

**Figure 4:**
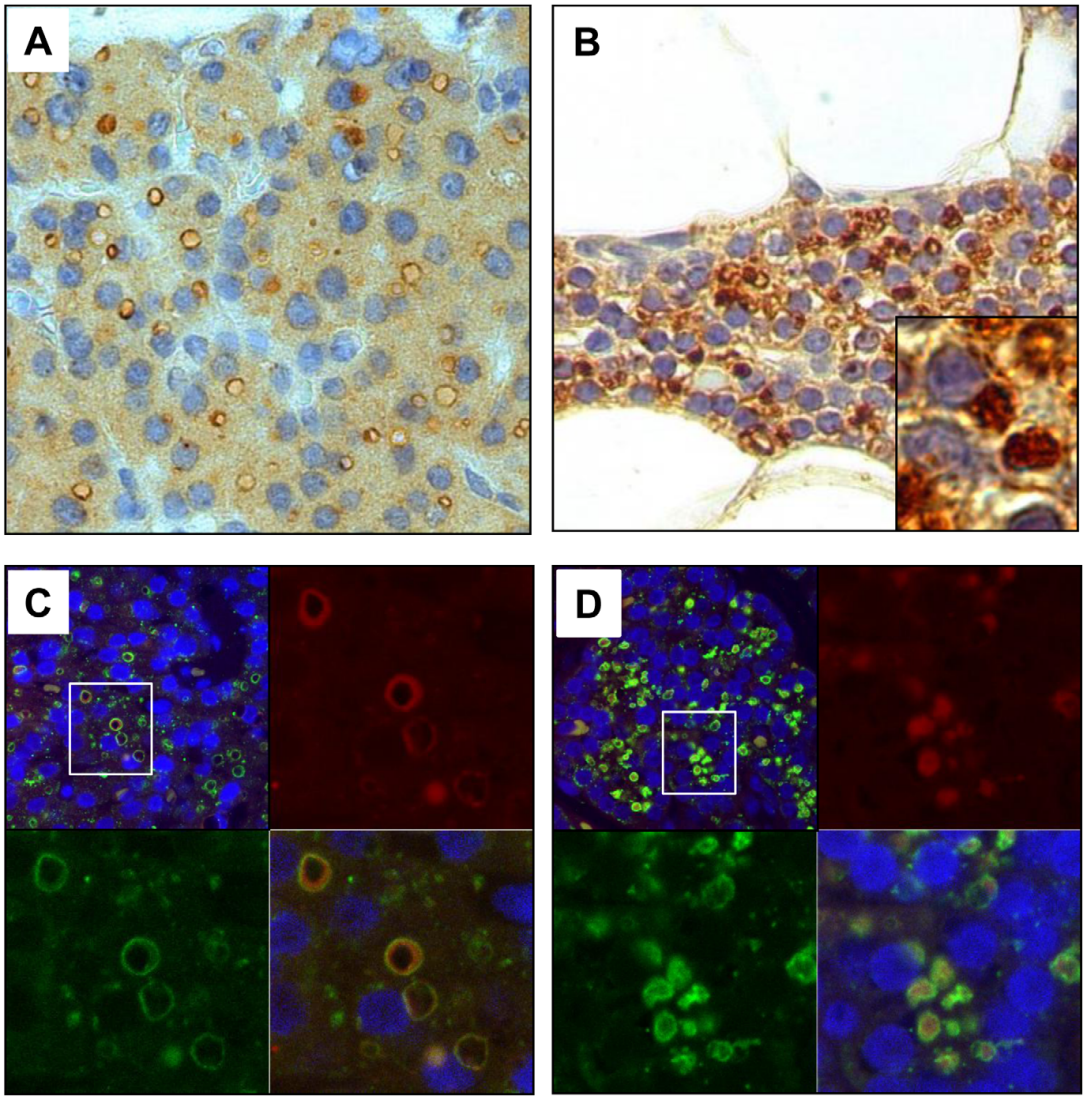
Localisation of PRLr expression to lysosomes in normal parathyroid rim and to enlarged lysosomes in parathyroid tumour tissue. A) Immunohistochemistry with PRLrI showing “ring-like” cytoplasmic structures and cytoplasmic reactivity. B) Immunohistochemistry of PRLrI showing cytoplasmic granuale and cytoplasmic reactivty. C and D) Analysis of “ring like” structures and cytoplasmic granulae by flourescent immunohistochemistry. Images show one parathyroid tumour (C) and normal parathyroid rim (D), stained with DAPI (blue), anti-PRLrI (red, upper right) and anti-SCARB2 (green lysosomal marker, lower left) separately and in overlay (lower right and upper left).

As different isoforms of the PRLr have been shown to be differentially expressed and localized to diverse parts of the cell in various tumours [41], [42], we aimed to characterize the sub-cellular localization as well as the overall expression of the PRLr using immunohistochemistry. Overall, the immunohistochemical results support our Western blot data suggesting that PRLr is expressed in the vast majority of all parathyroid tumours investigated. Using the PRLrI antibody, positive immunoreactivity was observed in all tumours analysed (n = 36), as well as in non-tumour parathyroid cells located in the normal rim that was present in the majority of parathyroid tumour sections (n = 28). Analysis of the subcellular localization revealed strong immunostaining in the cytoplasm and cytoplasmic granulae of all normal rims. Nuclear staining was never noted (Figure 3A). In contrast, several different staining patterns were revealed in parathyroid tumours, as illustrated in Fig. 3 and 4 (Table S3). Cytoplasmic expression of PRLr was observed in virtually all tumour cells, in 34/36 analysed cases, and 16 tumours showed immunostaining of cytoplasmic granulae in varying subsets of the cells. In addition, 12 tumours exhibited plasma membrane staining. Generally, staining of plasma membrane and granulae was not observed together in the same cell. In 4 cases staining of intracellular “ring-like” structures was observed (Figure 4A). In order to identify the cytoplasmic location giving rise to this phenomenon, fluorescence immunohistochemistry was done in two such cases with parallel analysis of anti-PRLr and markers for lysosomal or Golgi structures. In both cases, co-localization was observed in the tumour tissue for anti-PRLr and the lysosomal marker in the “ring-like” structures (Figure 4C), but not for the Golgi marker (data not shown), suggesting that they originate from PRLr localized to enlarged lysosomes. Furthermore, normal rim present in one of the cases showing PRLr expression in the cytoplasm and granulae (Figure 4B) revealed co-localization of anti-PRLr and the lysosomal marker in granulae suggesting a lysosomal origin of the granulae structures (Figure 4D).

A second antibody, PRLrA, was also evaluated by immunohistochemistry on the same cases (exemplified in Figure S4). In all normal rims analysed, immunoreactivity was observed in the cytoplasm and/or plasma membrane at varying intensities and proportions of the cells. Strong PRLr expression was detected in 24 tumours, while 5 had weak expression only and 7 were negative. In the 29 positive tumours immunoreactivity was located in both the cytoplasm and the plasma membrane (n = 16), or in either cytoplasm (n = 12) or the plasma membrane (n = 1). Focally different expression patterns were noted in 8 cases, while immunoreactivity was not evidently detected in nuclei, granulae or lysosomes.

### *PRLR*/PRLr Expression in Relation to Clinical Characteristics of Tumour Patients

Comparison of the prolactin receptor gene expression with clinical characteristics of the 37 parathyroid tumour cases revealed several significant relationships. Inverse correlations were observed between expression of *PRLR* and patients plasma calcium levels as demonstrated using both the *PRLR*-total (r = −0.333, P = 0.044), *PRLR*-LF1 (r = −0.336, P = 0.042) and the *PRLR*-LF2 (r = −0.345, P = 0.036) assays. The levels of *PRLR*-total were significantly lower for patients with calcium levels within the highest quartile (P = 0.033) and similar tendencies were found for *PRLR*-LF1, *PRLR*-LF2 and *PRLR*-S1a (P = 0.068; P = 0.061 and P = 0.053). With regard to the subcellular localization of PRLr, membranous immunostaining was not observed in cases with tumour weights in the highest quartile (P = 0.045).

### PTH Secretion and [Ca^2+^]_i_ at Prolactin Treatment of Parathyroid Adenomas

**Figure 5:**
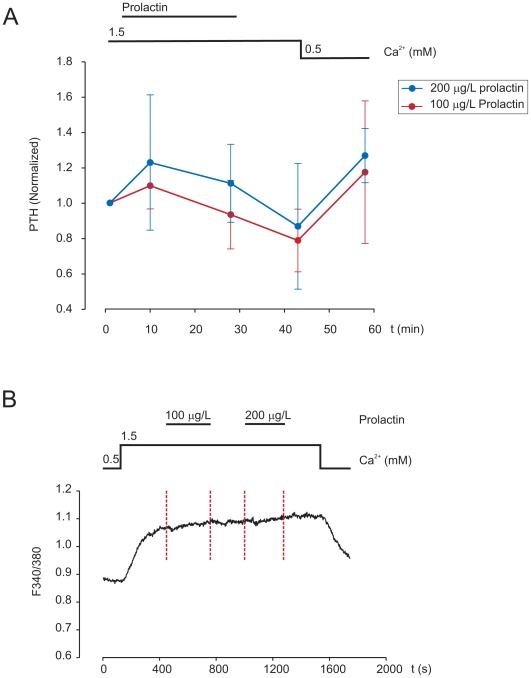
Analysis of PTH secretion and intracellular Ca^2+^ after prolactin treatment. A) Measurements of PTH secretion from parathyroid adenoma cells upon treatments with prolactin at 100 µg/L or 200 µg/L. Results from four independent experiments are shown. B) Example of a measurement of intracellular Ca^2+^ in parathyroid adenoma cells treated with 100 µg/L followed by 200 µg/L prolactin, as indicated by vertical red lines.

A possible effect on PTH secretion from prolactin stimulation of short-term cultured parathyroid tumour cells was studied by parallel perifusion at two different prolactin concentrations (100 or 200 µg/L). The findings are presented in Figure 5A. In summary, after 100 µg/L prolactin a 9.8±13.2% increase of PTH secretion, and after 200 µg/L prolactin a 22.8±38% rise in PTH secretion at 9 min was observed. Although the observations did not reach statistical significance, each individual experiment suggested an increase in PTH secretion. These observations indicate that prolactin may stimulate PTH secretion at a physiological concentration. Next, we investigated if [Ca^2+^]_i_ signalling was involved in the regulation using the Ca^2+^ indicator Fura-2. As shown in Figure 5B, addition of 100 µg/L or 200 µg/L prolactin did not significantly alter [Ca^2+^]_i_ levels.

### Gene Expression Patterns upon Prolactin Stimulation

**Figure 6:**
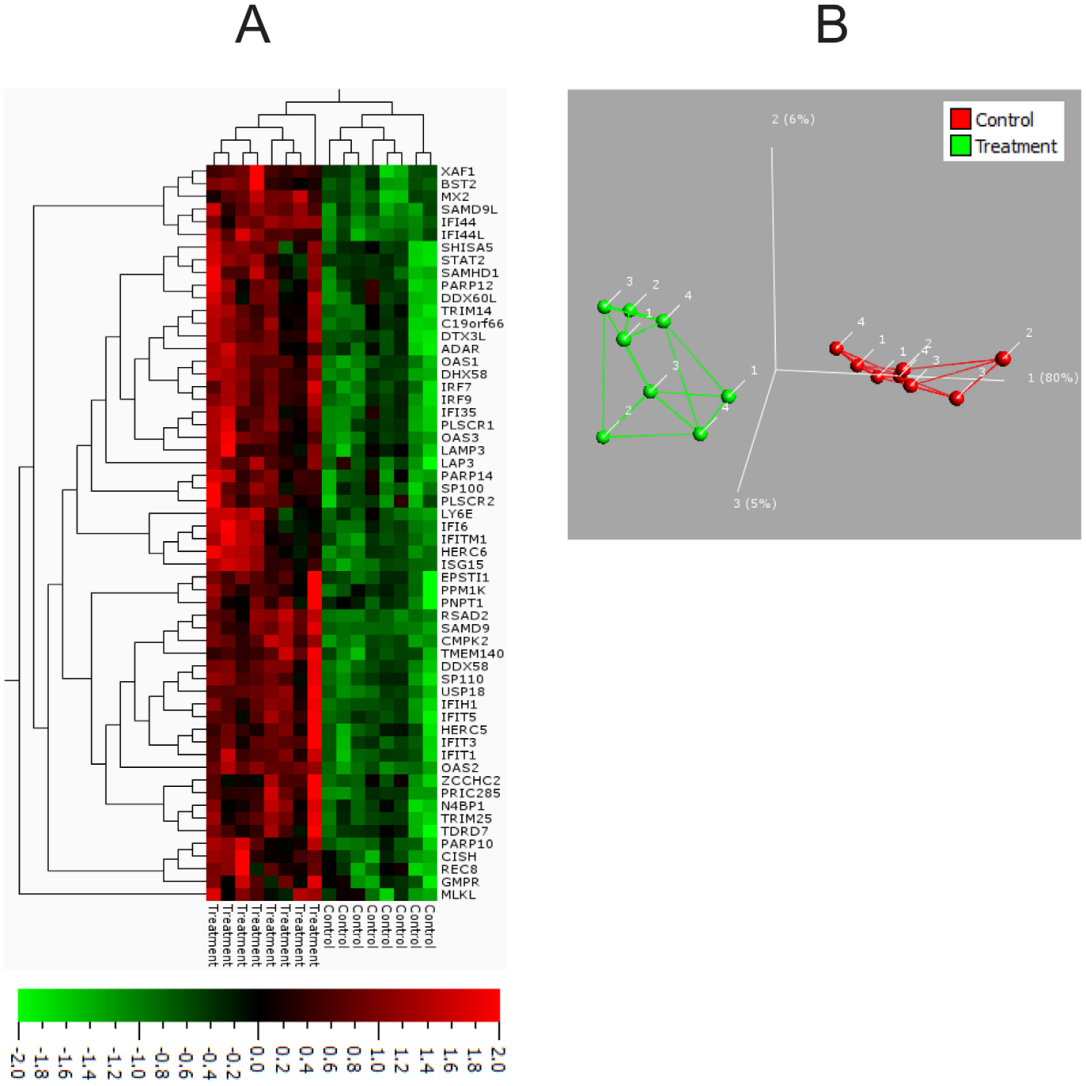
Clustering and PCA analysis of differentially expressed genes after prolactin treatment in parathyroid adenoma cells. A) Heat map of hierarchical clustering show 58 changed genes (adjusted P<0.01) as detected by Qlucore analysis. B) PCA of the gene expression data in a 2D graph, plotting individual primary cultures of parathyroid adenomas. Colours indicate either; green = control or red = prolactin treated. Numbers indicate individual cases. For significant genes (adjusted P<0.01), samples were grouped according to treatment. Lines indicate the three closest relationships to a sample.

Comparative gene expression profiling was done using the Affymetrix platform analysing parathyroid tumour cells treated with 200 µg/L prolactin as compared to untreated controls. After filtering of very low abundant probe sets, approximately 33,000 probe sets remained for further analysis. Paired t-test between treated and untreated tumours identified 63 differentially expressed probe sets (61 up-regulated and 2 down-regulated, Table S2). Pathway analyses identified statistically significant over-representation of dysregulated genes in the signalling pathways for retinoic acid inducible gene-I (RIG-I) like receptor-, JAK/STAT- and Type II interferon signalling pathways (Table 1). In addition a Qlucore analysis was performed. At PCA and clustering treated and control samples separated into two groups (Figure 6). The heatmap shows 58 significantly differentially expressed genes, all of which were upregulated. 44 of these genes overlapped with those detected by paired t-test and fold change cut-off (Table S2).

**Table 1:** Pathways with enrichment of dysregulated genes.

Pathway	Fold	Gene		Ensembl
Gene number	change	symbol	Gene name	gene ID
RIG-I-like receptor signaling (KEGG 04622)				
(C = 70; O = 6; E = 0.21; R = 28.27; rawP = 6.80e-08; adjP = 1.36e-07)				
23586	3.37	*DDX58*	DEAD (Asp-Glu-Ala-Asp) box polypeptide 58	ENSG00000107201
3665	3.14	*IRF7*	Interferon regulatory factor 7	ENSG00000185507
79132	2.19	*DHX58*	DEXH (Asp-Glu-X-His) box polypeptide 58	ENSG00000108771
64135	2.43	*IFIH1*	Interferon induced with helicase C domain 1	ENSG00000115267
7706	1.79	*TRIM25*	Tripartite motif-containing 25	ENSG00000121060
9636	2.54	*ISG15*	ISG15 ubiquitin-like modifier	ENSG00000187608
Jak-STAT signaling (KEGG 04630)				
(C = 153; O = 4; E = 0.46; R = 8.62; rawP = 0.0012; adjP = 0.0012)				
1154	1.78	*CISH*	Cytokine inducible SH2-containing protein	ENSG00000114737
6773	1.49	*STAT2*	Signal transducer and activator of transcription 2, 113 kDa	ENSG00000170581
6772	2.03	*STAT1*	Signal transducer and activator of transcription 1, 91 kDa	ENSG00000115415
10379	2.07	*IRF9*	Interferon regulatory factor 9	ENSG00000213928
Type II interferon signaling (Wikipathways WP619)				
(C = 50; O = 7; E = 0.15; R = 46.18; rawP = 1.49e-10; adjP = 1.49e-10)				
5610	2.27	*EIF2AK2*	Eukaryotic translation initiation factor 2-alpha kinase 2	ENSG00000055332
4938	7.71	*OAS1*	2',5'-oligoadenylate synthetase 1, 40/46 kDa	ENSG00000089127
2537	3.45	*IFI6*	Interferon, alpha-inducible protein 6	ENSG00000126709
6773	1.49	*STAT2*	Signal transducer and activator of transcription 2, 113 kDa	ENSG00000170581
9636	2.54	*ISG15*	ISG15 ubiquitin-like modifier	ENSG00000187608
6772	2.03	*STAT1*	Signal transducer and activator of transcription 1, 91 kDa	ENSG00000115415
10379	2.07	*IRF9*	Interferon regulatory factor 9	ENSG00000213928
C = Number of reference genes in the category; O = Number of genes in the gene set and also in the category.				
E = Expected number in the category; R = Ratio of enrichment.				
rawP = p-value from hypergeometric test; adjP = p-value adjusted by the multiple test adjustment.				

## Discussion

In this study we demonstrate high abundance of the prolactin receptor in parathyroid tissues, correlation of its expression levels to clinical characteristics, as well as localization and functional responses upon prolactin stimulation in parathyroid tumour cells.

Relatively high abundance of PRLr in parathyroid tissues was demonstrated at the gene transcripts and protein isoforms levels. The expression was compared with two cell lines frequently used as models for PRLr signalling in breast cancer: T47D and MCF-7 which are both known to express very high or high PRLr levels, respectively [43]. In Western blot analyses of parathyroid tissues, the 80 kDa PRLr product was detected at levels comparable to the T47D cell line, which is known to express the LF isoform [40]. Using qRT-PCR, parathyroid tissues were shown to have considerably higher *PRLR* expression compared to the MCF-7 cell line, which in turn had a higher level than the non-parathyroid normal tissues. In non-parathyroid tissues *PRLR* was most abundant in the placenta, followed by kidney, pancreas and lung, and lowest in brain, heart, lung and striated muscle, in agreement with previous observations by Peirce and Chen [43]. The findings were also extended to the analyses of individual transcripts, which showed similar results as the *PRLR*-total assay for simultaneous detection of the LF, ΔS1, IF and S1a transcripts. Hence the variation in *PRLR* observed in parathyroid tumours was not evidently related to the alteration of single transcripts.

GSK3β is able to phosphorylate ser349 of the prolactin receptor [24], a residue existing only in the long and ΔS1 isoforms. PRLr degradation is dictated by levels of GSK3β ser9-phosphorylation. The latter is in turn a known downstream target of menin/AKT signalling [44], [45]. As menin is a known primary affected tumour suppressor in parathyroid tumours, it is possible that its dysfunction would cause a disinhibition of GSK3β mediated degradation of PRLr, hence stabilizing the long/ΔS1 isoforms. This could to some extent explain the variation of long isoform expression in parathyroid tumours. If the same underlying mechanism is present in breast cancer, it would be an attractive target for treatment. Additionally, patients treated with lithium, which most likely leads to GSK3β phosphorylation [44], [46], are known to be highly prone to parathyroid tumours [47], [48], [49]. This connection remains to be investigated.

Comparison of parathyroid tumours and normal parathyroid tissues did not reveal differential levels of expression that were consistently reduced or increased as for a classical tumour suppressor or oncogene mechanism. Instead, we observed both increased and decreased levels of expression for total *PRLR*/PRLr and the various transcripts and isoforms. Interestingly, a statistically significant inverse correlation was observed between the *PRLR* gene expression and calcium levels, suggesting either a relationship to a subgroup of tumours and/or a causal change of calcium set point in the tumour.

PRLr expression showed frequent alteration of the subcellular signal location as compared to normal parathyroid rim. The PRLr expression pattern located to cytoplasm and granulae uniformly observed in normal rim was partly changed in parathyroid tumours. In the tumours the expression was cytoplasmic with additional location in cell membrane, granulae or enlarged lysosomes. Similar expression patterns with cytoplasmic granular and/or membranous location have been reported for other tumour types, where PRLr expression has also been associated with patient outcome [42]. In this study we similarly observed associations to patient disease phenotypes, both on protein and gene level. Specifically lack of membranous immunoreactivity was associated with larger adenomas (forth quartile). Still, in several parathyroid tumours membranous immunostaining was not detected, raising the question whether PRLr is still active in this subset of tumours. The association to clinical characteristics in parathyroid and other tumours would suggest a functional PRLr. Furthermore *in vitro* studies showed effects on gene expression patterns in parathyroid tumours. Taken together these results would suggest that PRLr is active but that the membranous level is too low for detection or not reachable by the antibody used in cases without strong membranous signal.

Functional effects on hormone secretion were evaluated upon prolactin stimulation of parathyroid adenoma cells. Although no statistically significant effect was documented, the results suggested that 200 µg/L PRL stimulate PTH-secretion in the acute phase in agreement with previous observations by Magliola *et al*. in bovine parathyroid cells [29]. However, in the bovine study prolactin exposure was done at 0.1–1.0 µM, which would correspond to supraphysiological levels. In healthy women prolactin levels are 7–10 µg/L under normal conditions, and rise to 75–100 µg/L during pregnancy and to 200–800 µg/L during lactation [32]. The present study suggests that PTH secretion may be acutely stimulated at physiological levels of prolactin. In respect to the observed correlation between *PRLR* gene expression and patient calcium levels, a study comparing responsiveness in high and low calcium phenotypes would be of interest.

In cultured parathyroid tumour cells, gene expression alterations upon prolactin stimulation affected RIG-I like receptor signalling, JAK-STAT signalling and Type II interferon signalling which were all increased. The JAK-STAT pathway is known to be activated by PRLr signalling [20]. Since PRLr is a cytokine receptor, the increase of Type II interferon signalling could be explained simply by PRLr engagement, or downstream co-activation of interferon-γ signalling by JAK2 crosstalk. A crosstalk between RIG-I like receptor signalling and PRLr signalling has not been described, suggesting that the upregulation cannot be explained by a direct crosstalk effect alone. However, given the common function of infectious response, a connection between the two would be an interesting object of study. Since no previous data regarding gene expression profiling and coupling to the PRLr in parathyroid tumours is known to be present, a direct comparison of our findings with independent observations could not be made. Some similarities between the effects observed in this study and published results in non-related tissues from knock-out mice do exist [20], thus supporting some of our main findings, such as JAK-STAT up-regulation upon prolactin stimulation. The addition of a non-parathyroid, prolactin-responsive control as a comparison could make an interesting backdrop to a future delineation of the PRLr receptor in human tumour systems.

The results obtained in this study suggest a functional role of PRLr in the parathyroid gland, and imply that PTH secretion may be stimulated acutely by prolactin. Further studies are required to evaluate the response to prolactin at different physiological and pathological states. Prolactin has been proposed to be the main calciotropic hormone during pregnancy, and is increased during lactation. In addition, high levels of prolactin may result from pathological conditions such as *e.g.* pituitary hyperfunction, polycystic ovary syndrome and iatrogenic hyperprolactinemia. Although this study does not conclude whether a physiological correlation exists or not, a response of PTH to physiological levels of prolactin is suggested. The earlier controversies regarding possible prolactin-PTH levels causality might be explained by the different dietary conditions of the subjects. For example, in North America and Europe PTH has been reported to decrease during pregnancy, while in areas of low or deficient dietary calcium (e.g. Brazil and Gambia), PTH was found to increase [30], [31], [50]. This difference may be a consequence of altered calcium recruitment from intestine to maternal bones. Increased risk of breast cancer has been associated with both high levels of prolactin [51] and PHPT [52]. Interestingly, an association between childbearing and PHPT has also been identified [53]. A logic continuation of the present study would be to evaluate a possible connection between prolactin levels and PHPT development.

In conclusion, we demonstrate high abundance of the prolactin receptor in the human parathyroid gland, aberrant subcellular localisation in parathyroid tumours, and varying levels of PRLr isoforms. Responsiveness to physiological levels of prolactin was observed in the form of increased PTH secretion within minutes of treatment, and altered gene transcription with significant increase of the RIG-I like receptor, JAK-STAT and Type II interferon signalling pathways. These data suggest a role of the prolactin receptor in parathyroid adenomas.

## Materials and Methods

### Ethics Statement

All parathyroid tumour samples and normal parathyroid tissues had been collected according to the established procedure at the Karolinska University Hospital as approved by the local ethical committee. Tissue samples were thus obtained with oral informed consent and documented in the patient files.

### Parathyroid Tumour Tissue Samples and Cell Cultures

Parathyroid tumours from 37 patients with PHPT and uniglandular disease were included in the study. Fresh-frozen tissue samples and sections from paraffin embedded tissue had been collected via the tissue biobank at Karolinska University Hospital. The panel of 37 cases was selected to represent the spectra of PHPT patients operated in the hospital during one year based on age at surgery (median 59 years; min-max 27–86), gender (30 female, 7 male), histopathological diagnosis and glandular weight (median 709 mg; min-max 230–13,500). Median total plasma calcium was 2.60 mmol/L (min-max 2.50–3.22), and median P-PTH was 145 ng/L (min-max 66–1,280). Tumours were diagnosed as adenoma (n = 33) or atypical adenoma (n = 4) in routine clinical histopathology according to WHO criteria [54]. Parathyroid adenoma samples for culturing and functional studies were obtained at surgery after informed consent. Up to one third of the tumour was collected from each sample, while the majority of the tissue was sent to routine histopathological diagnostics. Fresh tissue samples were quickly transferred in cold culturing media to the nearby laboratory, and then isolated and cultured using previously described methods [55]. All experiments were performed within 72 hours after isolation.

### Normal Samples

Normal non-tumour tissue of breast and fallopian tube samples from anonymized breast and ovarian cancer patients were fresh-frozen post-operatively. Normal parathyroid glands from normocalcemic patients were collected from patients operated for thyroid cancer with indication for parathyroid gland removal without re-implantation. All normal samples were stored at −80°C and verified by a histopathologist. Total protein extract and nuclear protein fraction of normal parathyroid tissue was commercially obtained (Cat. no. NB820-59245 and NBL1-26415; Novus Biologicals, Cambridge, UK). The MTC panel I purchased from Clontech (Cat. no. 636742, Clontech Mountain View, CA) includes cDNAs representing various normal tissues (normal brain, heart, kidney, liver, lung, pancreas, placenta and striated muscle). Two human breast carcinoma cell lines were used: Lysates of T47D were commercially obtained (Abcam, Cambridge, UK, cat. no ab14899); (Cat. no ab14899, Abcam, Cambridge, UK); and MCF-7 RNA extracts were kindly provided by Dr. Johan Hartman, Karolinska University Hospital.

### Quantitative Real-Time Polymerase Chain Reaction (qRT-PCR)

RNA was extracted using a commercially available kit (RiboPure TM Kit, Applied Biosystems, Carlsbad, CA), and concentrations were determined by Nanodrop ND-1000 (Nanodrop technology, Wilmington, DE). Quality was evaluated using an Agilent 2100 Bioanalyser (Santa Clara, CA), which showed that all included samples had a RIN value of >7.9. cDNA was synthesized with the High Capacity cDNA Reverse Transcription Kit (ABI) in 20 µl reactions with ∼15 µg/µl RNA and inclusion of RNAse inhibitor under recommended conditions (37°C for 30 min, 80°C for 30 min, final 4°C).

The PCR was run using 10 µl reaction mixtures per well in a 384-well plate and an ABI 7900HT Fast Real Time PCR System (ABI, Carlsbad, CA). The wells were loaded with equivalent amount of cDNA (1 or 2 µl of RT-PCR product or MTC panel I cDNA), 5 µl of Taqman universal mastermix II (ABI, Carlsbad, CA) and 0.5 µl of Taqman assay. Taqman assays from ABI were used including: *PRLR*-total (Assay on Demand Hs00168739_m1* detecting LF, ΔS1, IF and S1a), and *PRLR*-LF1 (detecting LF and ΔS1), *PRLR*-LF2 (detecting LF and ΔS1) and *PRLR*-S1a (detecting S1a) which were designed by ABI and bioinformatically analysed using software available online. *RPLP0* (Assay on Demand Hs99999902_m1) and *GAPDH* (Hs99999905_m1) were analysed in parallel as endogenous controls. All experiments included several negative controls where cDNA were exchanged by water.

For each assay, the qRT-PCR reaction products were separated by agarose gel electrophoresis to verify that a single product of the expected size was amplified. Relative expression was calculated using the ΔΔC_t_ method. Analysed samples had a C_t_<35. Outliers were automatically omitted from the analyses. Expression was normalized against the endogenous control *RPLP0* for parathyroid samples and against *GAPDH* for non-parathyroid normal tissues. The MCF-7 cell line was normalized against *RPLP0* or *GAPDH*. Relative quantification of gene expression was performed after normalization in relation to an arbitrary expression level of 1.0 assigned to the MCF-7 cell line, or to the mean for normal parathyroid samples. MCF-7 was chosen for comparison as it is known to express high levels of PRLr according to a previous publication by Peirce and Chen (42). All samples were run in triplicates or quadruplicates from which mean values of expression were calculated.

### Reverse-Transcription PCR (RT-PCR)

Possible expression of the *PRLR*-ΔS1 transcript was determined by RT-PCR using primers spanning selected exons followed by visualization of products in agarose gels containing GelRed (Life technologies, Delhi, India). Pooled cDNA samples from 4 parathyroid adenomas were used. cDNA (25 ng) was amplified with 2 µl primer (IDT, Coralville, Iowa) at 100 µM and 2 µl MgCl in 20 µl reactions under the conditions: 95°C for 10 min, 40×[95°C for 30 sec, annealing at 55.5–70.3°C for 30 sec, 72°C for 30 sec], and final 72°C. Primers for *PRLR*-ΔS1 were: Forward in exons 3–6 (5′-ATG TTC AGC CAG ACC CTC CT-3′); Reverse 1 in exon 7 (5′-GCA AAA TGG ATC TCC CAC TC-3′); Reverse 2 in exon 6 (5′-CTC CCA CTC AGC TGC TTT CT-3′); and Reverse 3 in exon 7 (5′-AAA TGG ATC TCC CAC TCA GC-3′). Primer sequences for detection of the other *PRLR* transcripts LF/ΔS1/S1a/S1b were: Forward in exon 3 (5′-ACC TGC CTT CTG AAT GGA C-3′); Reverse 1 in exon 5 (5′-TGT ACT GCT TGC CAA AGT-3′); Reverse 2 in exon 5 (5′-GGC CAC CGG TTA TGT AGT CT-3′); and Reverse 3 in exon 5 (5′-CCA CCG GTT ATG TAG TCT GGA-3′).

### Antibodies

Details of the monoclonal antibodies used (clone, manufacturer and working dilutions) are given in Table S1. The two PRLr antibodies target the extra-cellular domain corresponding to exons 3–7 (anti-PRLrI, Invitrogen) and an intracellular epitope of exon 10 (anti-PRLrA, AnaSpec), respectively (Fig. S1). The gPRLr antibody, specific for the N-glycosylated form of PRLr, targets the extra-cellular domain of exons 3–7. Anti-GSK3β targets total GSK3β, and the antibody for phosphorylated GSK3β is specific for the serine 9 phosphorylated form of GSK3β only. For fluorescent immunohistochemistry anti-GOLG1B and anti-SCARB1 served as subcellular markers of Golgi and lysosomal structures, respectively. Anti-GAPDH was used as a control of protein loading and quality for Western blot analysis.

The PRLr antibodies were assessed for PRLr sensitivity by dot blot analysis. Recombinant PRLr protein (0.1, 0.2 or 0.5 µg) for amino acids (aa) 432–622 in the intracellular domain was commercially obtained (Genway, San Diego, CA). The protein was applied to a nitrocellulose membrane, blocked for 1 hour with 5% milk in TBS-T at room temperature, incubated with the PRLrI or PRLrA antibodies, and developed as described for Western blot analysis below. Positive signal was observed for PRLrA, but not with PRLrI. No signal was observed with either antibody in the negative control experiments.

The PRLrA antibody was further verified by immunprecipitation. Dynabeads protein A (Invitrogen, Carlsbad, CA) were incubated with 2 µg of PRLrI antibody for 30 minutes at room temperature. 500 µg protein lysate from fallopian tube and parathyroid tumour (to cover all isoforms) were incubated with the antibody-bead-complex overnight under mild rotation at 4°C. After resuspension in elution buffer, the beads were separated using a magnet-rack. Immunoprecipitated samples were analysed by Western blot using PRLrI or PRLrA antibodies, which both revealed immunoreactivity against a 80 kDa product, corresponding to the long isoform of PRLr.

### Western Blot Analysis and Immunohistochemistry

Methodology for total protein extraction, Western blot analysis, and immunohistochemistry has been previously described [56], [57]. Immunohistochemistry was carried out on deparaffinized 4 µm tissue slides. Working conditions were optimized for different heat induced antigen retrieval techniques and antibody dilutions. Antigen retrieval techniques included citrate- and EDTA-buffer (heated 0, 10 and 20 minutes for each solution respectively), and antibody dilution ranges were based on previous data and recommended concentrations. For antigen retrieval, the slides were treated with 95°C citrate buffer pH 6.0 (Dako, Glostrup, Denmark) for 20 minutes in a microwave oven. Endogenous biotin was neutralized using the Avidin-Biotin Blocking Kit (SP-2001, Vector Laboratories, Burlingame, CA, USA). Slides were incubated with a primary antibody (Table S1) diluted in 1% BSA at 4°C overnight followed by the horse anti-mouse biotinylated secondary antibody diluted at 1∶700 (BA-2000, Vector Laboratories) for 45 minutes in room temperature, followed by Avidin-biotin-peroxidase complex (Vectastain Elite Kit, Vector Laboratories, CA, USA) incubation for 45 minutes and diaminobenzidine tetrahydrochloride for 6 minutes. Haematoxylin was used as counterstaining. For negative controls, the primary antibody was omitted. Negative controls were run in parallel to all experiments. Slides were evaluated and photographed using a Zeiss Axioskop (Carl Zeiss, Jena, Germany) equipped with Zeiss Plan-Neofluar lenses, and a ProgRes C12 Plus camera, and ProgRes Capture Pro 2.5 software (Jenoptik, Jena, Germany). The immunostaining was evaluated by four of the authors (F.H., C.J., C.L., A.H), concerning expression and subcellular localisation, in tumour and adjacent normal tissue if available. A subset of slides were scanned in a slide scanner (Hamamatsu, Shizuoka, Japan) and analyzed with NDP view software (Hamamatsu, Shizuoka, Japan).

### Fluorescence Immunohistochemistry

Paraffin embedded tissue slides from three parathyroid adenomas were analysed by fluorescence immunohistochemistry. Primary antibodies: PRLrI, anti-SCARB2 (lysosymal marker) and anti-GOLGB1 (Golgi marker) and fluorescent secondary antibodies: Fluorescent anti-rabbit Alexa Fluor 488 (Abcam, Cambridge, UK) and fluorescent anti-mouse Alexa Fluor 546 (Abcam, Cambridge, UK) were used for demonstrating the presence and localization of the investigated proteins in tissue sections, Images were obtained by Confocal Laser Scanning Microscopy (CLSM), using a uniquely modified ConfoCor3 instrument (Carl Zeiss, Jena, Germany) consisting of an inverted microscope for transmitted light and epifluorescence (Axiovert 200 M), a VIS-laser module comprising the Ar/ArKr (458, 477, 488 and 514 nm), HeNe 543 nm and HeNe 633 nm lasers and the LSM 510 META module. The instrument was modified to enable detection using silicon avalanche photodiodes (APD; SPCM-AQR-1X, PerkinElmer, USA) for imaging. Triple fluorescence images were recorded using a standard HBO103 mercury lamp for DAPI (blue pseudocolor), the 488 nm line of the Ar/ArKr laser for Alexa Fluor 488 (green pseudocolor) and the 543 nm laser line for Alexa Fluor 546 (red pseudocolor). DAPI fluorescence was acquired under bright field illumination and point scan detection using a photomultiplier tube (PMT), whereas Alexa Fluor 488 and 546 signals were acquired under confocal setting using APD for signal detection. Fluorescent signals were separated using the NFT 490 and HFT 488/543 beam splitters, and the BP 390-465 IR, BP 505-530 IR and LP 655 filters. The C-Apochromat 40×/1.2 W UV-VIS-IR objective was used throughout. Images were recorded at 512×512 pixel resolution, without averaging, scanning speed 25.6 µs/pixel.

### Measurement of PTH Secretion by Perifusion

Perifusion analyses of PTH secretion were performed essentially as previously described [55]. Cells were first suspended in medium overnight to recover from collagenase digestion and analysed by Trypan blue methodology to confirm high cell viability (>98%). Cells (1×10^4^–1×10^5^) were subsequently loaded into a perifusion column with P-4 gel, which was closed and kept at 37°C. Perifusion was effectuated with a peristaltic pump at a speed of 500 µl/3 min. Before each experiment, preperifusion was done for 30 min with extracellular solution (EC), 1.5 mM Ca^2+^ and basal amino acid. Prolactin treatment included 9 min perifusion with 1.5 mM Ca^2+^, addition of prolactin (recombinant human prolactin, Cat. no JM-4687-50, MBL Woburn, MA) for 18 min, and washout. To verify cell viability after the experiment, 0.5 mM Ca^2+^ was administered to the column. Samples were collected every 3 min, stored at −20°C, and quantified for intact PTH at the routine clinical chemistry laboratory in the Karolinska University Hospital, Stockholm, Sweden (ISO 15189 certified). To increase the comparability, effect of 100 µg/L and 200 µg/L prolactin were tested in parallel using cells from the same adenoma. Each protocol was performed four times with cells from four different adenoma glands. To allow comparison, the initial PTH level in each experiment was set to the arbitrary value of 1, and the PTH levels were normalized accordingly.

### Measurement of Intracellular Ca^2+^ ([Ca^2+^]_i_) by Fura-2

Measurements of [Ca^2+^]_i_ were carried out following procedures previously described in detail by Lu *et al*. [55] In brief, isolated cells were grown overnight on glass cover slips to enable attachment. The cover slips were placed in a perifusion chamber at 37°C and loaded with 2.5 µM Fura-2 AM (Invitrogen) in extracellular (EC) perifusion solution supplemented with 1.25 mM CaCl_2_. The cells were stepwise stimulated with 0.5 or 0.1 mM Ca^2+^ and 1.5 mM Ca^2+^ followed by addition of prolactin at 100 or 200 µg/L. Fluorescence was collected through an inverted fluorescence microscope equipped with an 40× oil immersion objective, and recorded using a cooled charged-coupled device (CCD) camera connected with an imaging system. Fura-2 fluorescence was alternately excited at 340 nm and 380 nm. In either case, fluorescence emission at 505 nm was recorded. Fluorescence intensities ratio 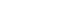 was used to represent the [Ca^2+^]_i_ level. Four independent experiments were performed on at least three patient samples.

### Expression Profiling

Expression profiling was done in parathyroid adenomas subjected to prolactin treatment in culture. In addition, corresponding paraffin sections were obtained for verification of PRLr expression by immunohistochemistry. 200 µg/L prolactin (recombinant human prolactin, Cat. No. JM-4687-50, MBL Woburn, MA) was added to 1×10^6^ attached parathyroid tumour cells. Cells were harvested using RNAlater (QIAGEN) and homogenized with QIAshredder for RNA extraction after 3 h and 24 h in culture, respectively. Negative controls were collected in parallel with each case at the same time points. RNA was extracted using QIA Cube, and quality assessed with Bioanalyser and Nanodrop. Expression array profiling and data analysis was done at the KI core facility Bioinformatics and Expression Analysis (BEA, Novum, Huddinge) using the Affymetrix platform and the TITAN ST 1.1 array. In short; Ambion WT Expression kit was used for total RNA conversion to sense strand cDNA, fragmentation and labelling was accomplished with WT GeneChip WT target Labeling kit. GeneTitan Hybridization, Wash and Stain Kit for WT Array Plates were used for hybridization to Affymatrix Human Gene 1.1 ST Array Plates. Plates were scanned using Affymatrix GeneChip HT Array Plate Scanner. Pre-processing of data was conducted with the Affymatrix Expression Console using the following methods: Summarization: PLIER, Background: Correction: PM-GC-BG, Normalization: Global Median. Value definition: Untransformed signals. A total of 16 samples were analysed including 4 parathyroid adenomas cultured for 3 h or 24 h in the presence of prolactin plus control samples cultured in parallel without prolactin.

Post process data analyses were performed as follows. After normalization, probe sets with mean expression values below 30 in both treated and untreated sample groups were excluded. In order to detect both early and late genes, samples were grouped into treated (n = 8) and untreated (n = 8) cells. Paired t-test was performed for comparative analysis to exclude non-significant genes. Individual gene inclusion criteria included; P-value of <0.01 and fold change of < −1.4 or >1.4. Filtered genes were analysed by WebGestalt (http://bioinfo.vanderbilt.edu/webgestalt/option.php) for enrichment analysis (KEGG and Wikipathways by hypergeometric distribution, cut-offs included a minimal significance level of P<0.02 and minimum 4 genes) and gene ontology (GO) classification. All microarray data are available at NCBI’s Gene Expression Omnibus, and are available through accession number GSE32387, or http://www.ncbi.nlm.nih.gov/sites/GDSbrowser?acc=GDS32387. For PCA plot and Heatmap generation, pre-processed data was analysed in Qlucore (Qlucore AB, Lund Sweden). Data were corrected nominally for individual dependency with an additional variance filtering of 7e-4. We employed a two-group comparison, two sided t-test with a adjusted P-value cut-off of <0.01.

### Statistical Analyses

All statistical calculations of clinical data were performed using the IBM SPSS software (Statistical Software Package for Windows, V.20). Data was analysed with the Pearson Chi-Square test for qualitative variables and Mann-Whitney U test for continuous variables. Relationships between variables were assessed with Spearman’s rank correlation test. P-values <0.05 were taken as statistically significant.

## Supporting Information

Figure S1 **Schematic illustration of the mRNA transcripts and corresponding protein isoforms for the prolactin receptor gene locus.** Location of qRT-PCR assays are indicated at the top, approximate protein sizes to the left and location of antibody epitopes and GSK3β interaction site below. LF = Long form, ΔS1 = delta S1, IF = intermediate form, S1a = short form 1a, S1b = short form 1b.
(TIF)

Figure S2 **Results from qRT-PCR analysis of the** ***PRLR*** **gene in individual samples of normal tissues as compared to the MCF-7 cell-line.** The column charts show results from the assays *PRLR*-total (for LF, ΔS1, IF and S1a), *PRLR*-LF1 (for LF and ΔS1), *PRLR*-LF2 (for LF and ΔS1) (A); and the assay *PRLR*-S1a for the S1a transcript only (B). The arbitrary expression level of 1.0 indicates the expression level for MCF-7 cells.
(TIF)

Figure S3 **Results from qRT-PCR analysis of the** ***PRLR*** **gene in individual samples of parathyroid tumours and normal parathyroids.** The column charts show results from the assays *PRLR*-total (for LF, ΔS1, IF and S1a), *PRLR*-LF1 (for LF and ΔS1), *PRLR*-LF2 (for LF and ΔS1), and *PRLR*-S1a (for S1a). The arbitrary expression level of 1.0 indicates the mean expression value for normal parathyroids.
(TIF)

Figure S4 **Immunohistochemical analysis of PRLr expression in normal rim and three different parathyroid tumours using the PRLrA antibody.** A) In normal rim immunoreactivity is observed in cytoplasm and/or plasma membrane. In the tumours PRLr expression is localized in plasma membrane and cytoplasm (B), in cytoplasm (C), and in plasma membrane and cytoplasm (D).
(TIF)

Table S1 **Details of antibodies used in this study.**
(XLS)

Table S2 **Differentially expressed probe sets/genes in prolactin treated vs. untreated parathyroid tumors.**
(XLS)

Table S3 **Gene expression and subcellular localization of the prolactin receptor in parathyroid tumor panel.**
(XLS)
