# Supplementary figures and images for: Prolactin Receptor in Primary Hyperparathyroidism – Expression, Functionality and Clinical Correlations
Source: PLoS One. 2012 May 11;7(5):e36448. doi: 10.1371/journal.pone.0036448 (PMC3350524; doi:10.1371/journal.pone.0036448)

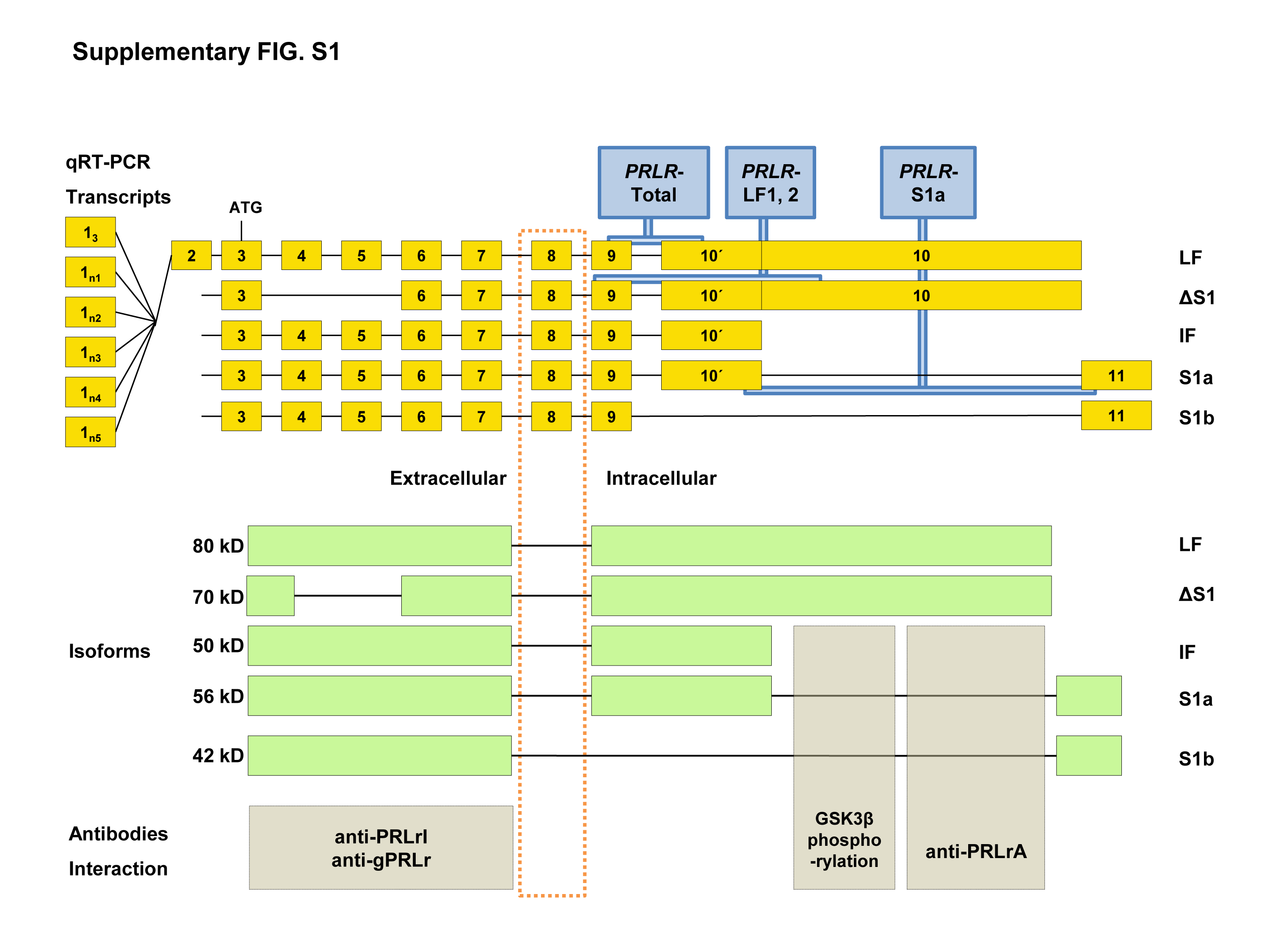

Supplement: Figure S1 — Schematic illustration of the mRNA transcripts and corresponding protein isoforms for the prolactin receptor gene locus. Location of qRT-PCR assays are indicated at the top, approximate protein sizes to the left and location of antibody epitopes and GSK3β interaction site below. LF = Long form, ΔS1 = delta S1, IF = intermediate form, S1a = short form 1a, S1b = short form 1b. (TIF) [file pone.0036448.s001.tif]

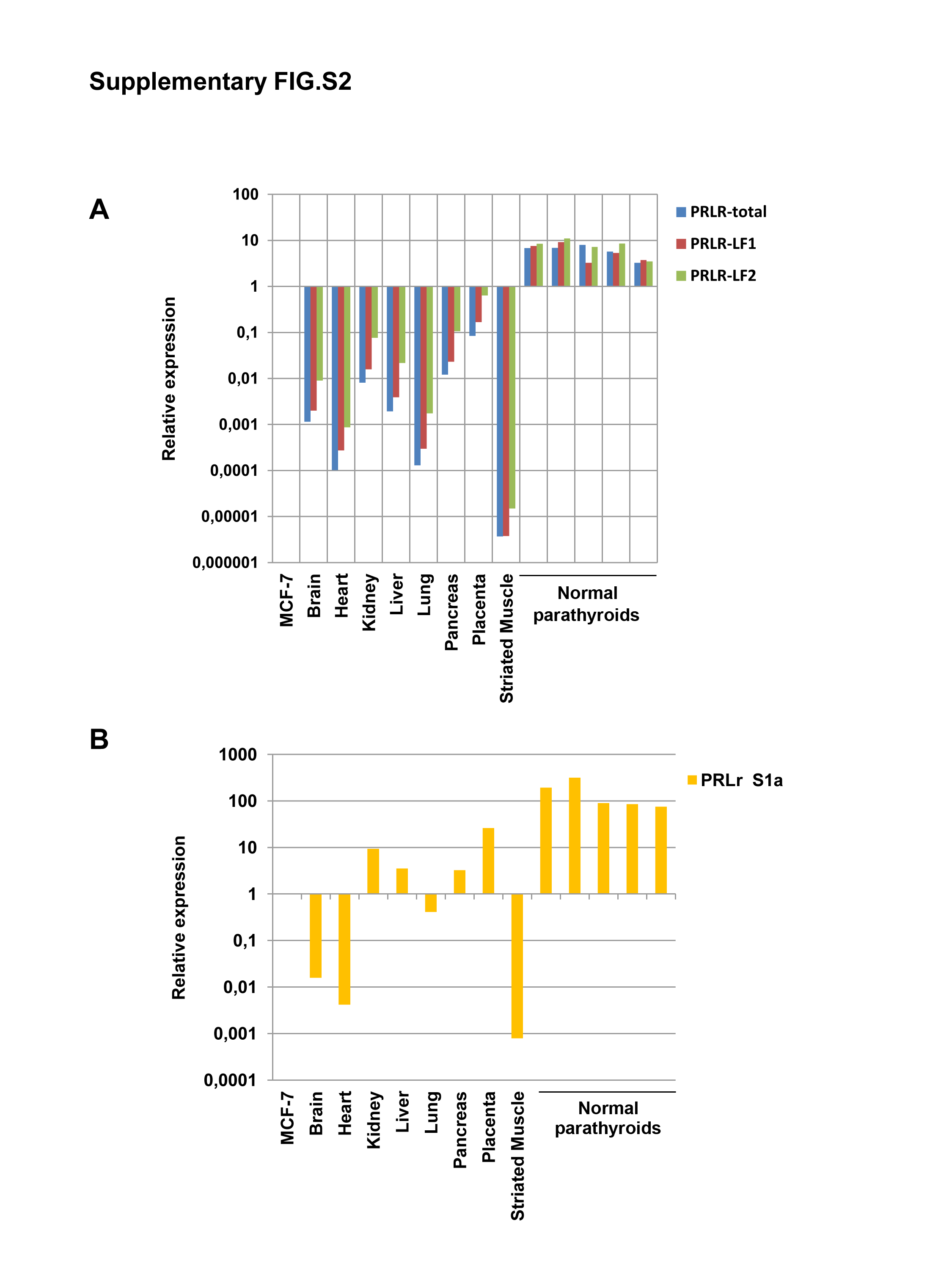

Supplement: Figure S2 — Results from qRT-PCR analysis of the PRLR gene in individual samples of normal tissues as compared to the MCF-7 cell-line. The column charts show results from the assays PRLR-total (for LF, ΔS1, IF and S1a), PRLR-LF1 (for LF and ΔS1), PRLR-LF2 (for LF and ΔS1) (A); and the assay PRLR-S1a for the S1a transcript only (B). The arbitrary expression level of 1.0 indicates the expression level for MCF-7 cells. (TIF) [file pone.0036448.s002.tif]

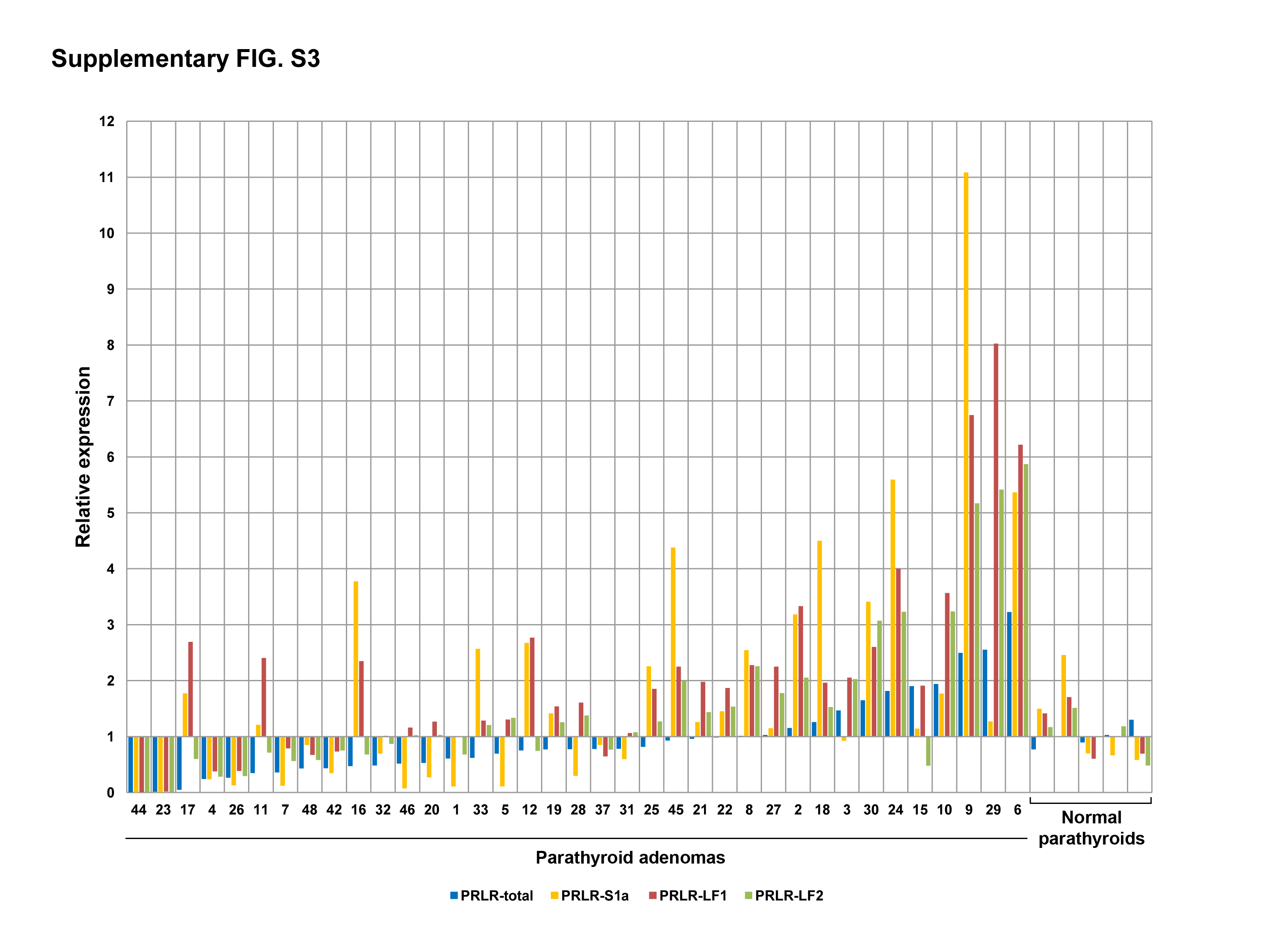

Supplement: Figure S3 — Results from qRT-PCR analysis of the PRLR gene in individual samples of parathyroid tumours and normal parathyroids. The column charts show results from the assays PRLR-total (for LF, ΔS1, IF and S1a), PRLR-LF1 (for LF and ΔS1), PRLR-LF2 (for LF and ΔS1), and PRLR-S1a (for S1a). The arbitrary expression level of 1.0 indicates the mean expression value for normal parathyroids. (TIF) [file pone.0036448.s003.tif]

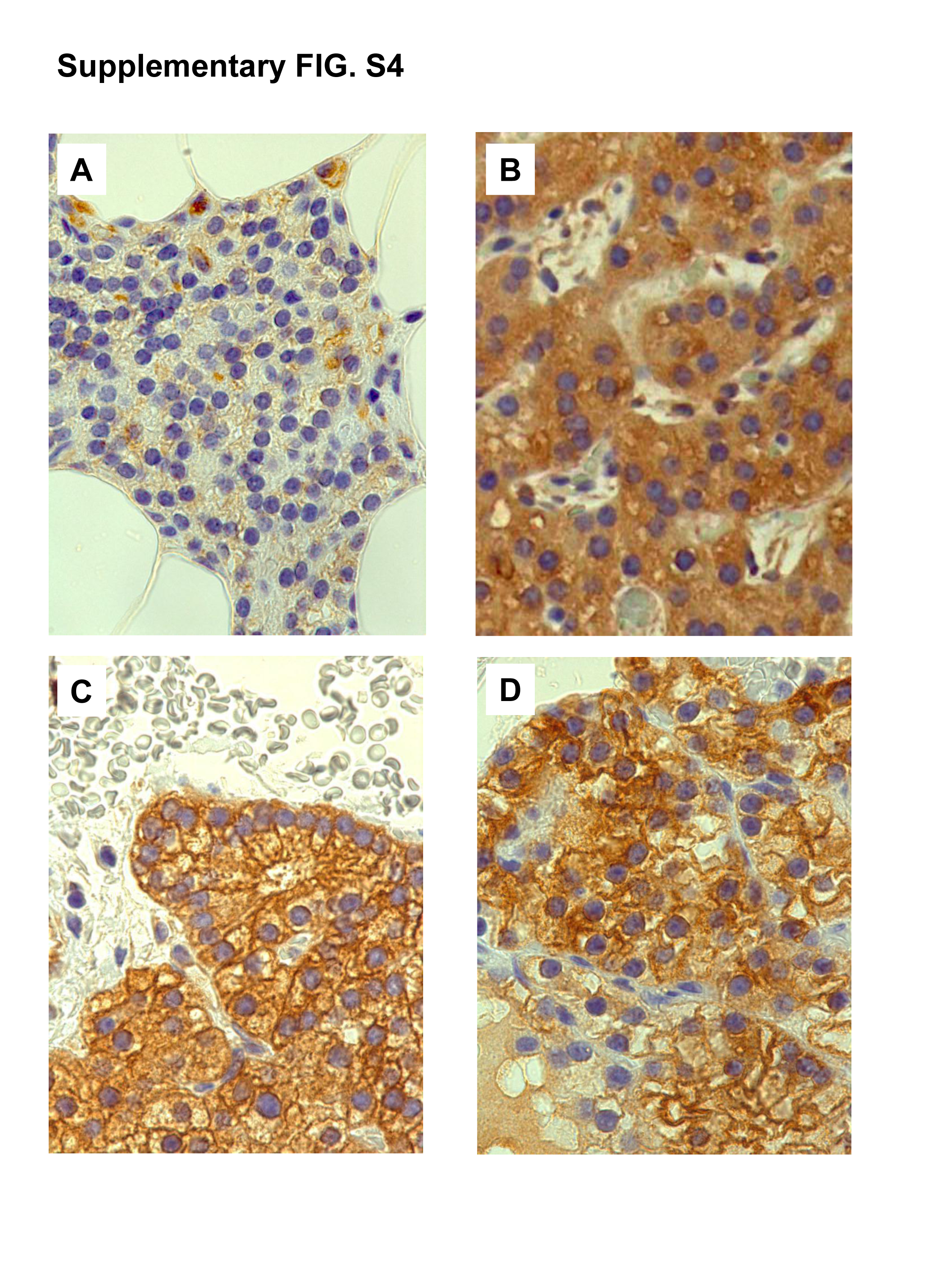

Supplement: Figure S4 — Immunohistochemical analysis of PRLr expression in normal rim and three different parathyroid tumours using the PRLrA antibody. A) In normal rim immunoreactivity is observed in cytoplasm and/or plasma membrane. In the tumours PRLr expression is localized in plasma membrane and cytoplasm (B), in cytoplasm (C), and in plasma membrane and cytoplasm (D). (TIF) [file pone.0036448.s004.tif]
